# Engineering the phototropin photocycle improves photoreceptor performance and plant biomass production

**DOI:** 10.1073/pnas.1902915116

**Published:** 2019-06-03

**Authors:** Jaynee E. Hart, Stuart Sullivan, Paweł Hermanowicz, Jan Petersen, L. Aranzazú Diaz-Ramos, David J. Hoey, Justyna Łabuz, John M. Christie

**Affiliations:** ^a^Institute of Molecular, Cell and Systems Biology, College of Medical, Veterinary, and Life Sciences, University of Glasgow, G12 8QQ Glasgow, United Kingdom;; ^b^Laboratory of Photobiology, Malopolska Centre of Biotechnology, Jagiellonian University, 30-387 Krakow, Poland;; ^c^Department of Plant Biotechnology, Faculty of Biochemistry, Biophysics, and Biotechnology, Jagiellonian University, 30-387 Krakow, Poland

**Keywords:** adduct decay, biomass, LOV domain, phototropin, photosynthetic efficiency

## Abstract

The ability to enhance photosynthetic capacity remains a recognized bottleneck to improving plant productivity. Phototropin blue light receptors (phot1 and phot2) optimize photosynthetic efficiency in *Arabidopsis thaliana* by coordinating multiple light-capturing processes. In this study, we explore the potential of using protein engineering to improve photoreceptor performance and thereby plant growth. We demonstrate that targeted mutagenesis can decrease or increase the photocycle lifetime of *Arabidopsis* phototropins in vitro and show that these variants can be used to reduce or extend the duration of photoreceptor activation *in planta*. Our findings show that slowing the phototropin photocycle enhanced several light-capturing responses, while accelerating it reduced phototropin’s sensitivity for chloroplast accumulation movement. Moreover, plants engineered to have a slow-photocycling variant of phot1 or phot2 displayed increased biomass production under low-light conditions as a consequence of their improved sensitivity. Together, these findings demonstrate the feasibility of engineering photoreceptors to manipulate plant growth and offer additional opportunities to enhance photosynthetic competence, particularly under suboptimal light regimes.

## Significance

A key challenge for plant molecular biologists is to increase plant yield by altering photosynthetic productivity to secure food, energy, and environmental sustainability. In the model plant *Arabidopsis thaliana*, the plasma-membrane–associated phototropin kinases, phot1 and phot2, are activated by blue light and play important roles in regulating several responses that optimize photosynthetic efficiency. However, little effort has been made to target these pathways to increase plant growth. Here, we demonstrate that modifying the photocycle of phot1 and phot2 increases their sensitivity to light. Plants with these engineered phototropins exhibit more rapid and robust chloroplast movement responses and improved leaf positioning and expansion, leading to improved biomass accumulation under light-limiting conditions.

Enhancing the efficiency of photosynthesis has the potential to increase plant biomass and improve crop yield. To date, the engineering strategies used to achieve this have centered on manipulating the reaction processes that underlie photosynthesis (1). These include minimizing energy loss during light absorption and accelerating carbon fixation (2). Additional increases in productivity, above those that can be achieved by these approaches, will depend on the development of alternative strategies to enhancing photosynthetic productivity.

Phototropins are plasma membrane-associated kinases that are activated by blue light (BL) (3). Flowering plants, including *Arabidopsis*, contain two phototropins (phot1 and phot2) (4, 5), which play important roles in regulating leaf positioning and expansion, chloroplast movements, stomatal opening, and phototropism, all of which serve to optimize photosynthetic efficiency (6). Thus, the modulation of phototropin activity could offer new opportunities to enhance photosynthesis by improving the efficiency of light capture via multiple physiological responses. However, as yet, no attempts have been made to engineer the phototropins to improve their activity.

Phototropins contain a serine/threonine kinase domain at their C terminus and two light-, oxygen-, or voltage-sensing domains (LOV1 and LOV2) at their N terminus. Both LOV1 and LOV2 function as BL sensors and bind flavin mononucleotide (FMN) as chromophore (7), yet they exhibit distinct roles in regulating phototropin activity (8–10). LOV2 plays a major role in regulating light-dependent receptor autophosphorylation (8, 9, 11), a process that is essential for phototropin function (12, 13), whereas LOV1 is believed to modulate the action of LOV2 (10, 14) and to mediate receptor dimerization (15).

The structural and biophysical properties of LOV domains have been well characterized (6). This domain primarily consists of five antiparallel β-sheets and two α-helices in which the FMN chromophore is bound noncovalently inside the β-scaffold (16). Photoexcitation produces a flavin triplet state (17) that leads to the formation of a covalent adduct between the FMN isoalloxazine ring and a conserved cysteine residue within the LOV domain (18, 19). Adduct formation triggers photoreceptor activation by invoking the side chain rotation of a nearby glutamine residue; this rotation is required to propagate structural changes at the β-sheet surface (20, 21). These structural changes also affect helical segments that flank the N and C terminus of the LOV2-core (22, 23), which are proposed to repress phototropin kinase activity in darkness. BL, or the artificial disruption of these helical segments by targeted mutagenesis, abrogates this repression (24–26), leading to receptor autophosphorylation (6).

LOV domains are versatile photosensory modules that can regulate a diverse range of biological outputs in plants, fungi, and bacteria (27). Light-induced adduct formation is reversible in darkness and thermally decays back to the ground state within tens to thousands of seconds, depending on the LOV domain involved (16). This broad spectrum in adduct decay kinetics has opened up new possibilities to modulate the action of LOV-based photoreceptors. Indeed, random and structure-based mutagenesis approaches have successfully identified single amino acid changes that can alter the rate of adduct decay (28–30). For example, the adduct state of the fungal LOV photoreceptor Vivid (VVD) is very stable (*t*_1/2_ ∼ 5 h) but can be accelerated by over four orders of magnitude (30, 31). Conversely, the slowing of the photocycle of the LOV-containing protein Zeitlupe (ZTL) has successfully altered the rate of clock-component degradation, thereby impacting the circadian period in *Arabidopsis* (32). This tunable aspect of the LOV photocycle has also been key to establishing it as an integral component of the optogenetic toolkit (16, 33).

The aim of this study was to determine whether targeted engineering can modify the reactivity of *Arabidopsis* phot1 and phot2 to enhance their responsiveness and to promote plant growth. We report here the identification of amino acid substitutions within the LOV2 photosensory module that can speed up or slow down adduct decay. We demonstrate that these variants can alter phototropin sensitivity *in planta* and that slow-cycling variants in *Arabidopsis* exhibited enhanced responsiveness that translated into increased plant growth under light-limiting conditions. These findings thus highlight the potential for protein engineering strategies to fine-tune phototropin sensitivity to improve plant photosynthetic capacity and growth.

## Results and Discussion

### LOV2 Mutagenesis Can Accelerate or Slow Down Phot1 Adduct Decay.

**Fig. 1.**
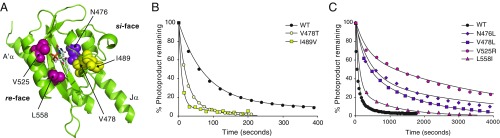
Amino acid substitutions that impact phot1 adduct decay. (*A*) Structure of *Arabidopsis* phot1 LOV2 presented as ribbons (PDB ID code 4HHD). Amino acid residues targeted for mutagenesis are labeled with arrows, along with A′α- and Jα-helices. FMN is presented as a ball-and-stick model with red oxygen and blue nitrogen atoms. (*B*) Adduct decay kinetics for *Arabidopsis* phot1 LOV1+2 (WT) and for the fast-cycling variants V478T and I489V. Purified proteins were irradiated with a WL camera strobe flash and adduct decay was recorded at an absorbance of 450 nm. Decay is expressed as percentage of photoproduct remaining and calculated from the ratio of absorbance at 450 nm obtained immediately after light treatment to that obtained for subsequent sampling in darkness. (*C*) Adduct decay kinetics for phot1 LOV1+2 (WT) and slow-cycling variants N476L, V478L, V525R, and L558I. Adduct decay for WT shown in *A* is included for reference.

A truncated fragment of *Arabidopsis* phot1 that consists of both LOV domains (LOV1+2) has similar photochemical properties as the full-length receptor protein (34). We therefore used this truncated phot1 to assess the impact of candidate LOV2 photocycle mutations on adduct decay kinetics (Fig. 1*A*). Light-induced adduct formation for phot1 LOV1+2 coincides with a loss of absorption at 447 nm (*SI Appendix*, Fig. S1*A*) that fully recovers on a time scale of ∼15 min in darkness (Fig. 1*B*).

Directed evolution combined with fluorescence-based imaging in *Escherichia coli* has been successfully used to isolate both fast- and slow-photocycling variants of LOV2 (28, 29). For example, the I427V substitution in *Avena sativa* phot1 decreased the lifetime of the LOV2 adduct state by one order of magnitude (28). Incorporation of the equivalent substitution, I489V (Fig. 1*A*), into *Arabidopsis* phot1 was also successful in accelerating adduct decay (Fig. 1*B*). A second fast-cycling variant, V478T (29) (Fig. 1*A*), was similarly effective at accelerating phot1 adduct decay (Fig. 1*B*). Conversely, mutation of V478 (Fig. 1*A*) to leucine has been reported to slow the photocycle of LOV2 (29). Consistent with these findings, we found that introducing the V478L (Fig. 1*C*) or the V478I mutation (*SI Appendix*, Fig. S2) into *Arabidopsis* phot1 slowed the rate of adduct decay, as did the mutation of N476 (Fig. 1*A*) to leucine, which has also been reported to attenuate the LOV2 photocycle (35).

Two arginine residues that interact with the FMN chromophore and that are absent from the phototropin LOV domains have been attributed to the very slow photocycle kinetics (*t*_1/2_ ∼ 29 h) of the LOV protein PpSB1 from *Pseudomonas putida* (36, 37). Substituting one of the equivalent residues in phot1, V525 (Fig. 1*A*), with arginine was very effective in extending the lifetime of the adduct state (Fig. 1*C*). Substitution of L558 (Fig. 1*A*) to isoleucine was also explored since a similar mutation in *Neurospora* VVD had been shown to slow its photocycle (30). Adduct decay in the L558I variant was found to be only moderately slower compared with that of the other slow photocycle mutations examined (Fig. 1*C*). Half-lives for adduct decay are summarized in Table 1.

**Table 1.** Half-lives for adduct decay obtained for the phot1 photocycle variants

Variant	*t*_1/2_
WT	51 s 495 s
V478T	13 s 38 s
V489I	6 s 101 s
N476L	289 s 2,310 s
V468L	154 s 1,155 s
V525R	217 s 2,311 s
L558I	67 s 693 s

In each case, decays were fitted to two exponentials.

Together, these findings demonstrate that the phot1 photocycle can be successfully altered by targeting residues at either the *si*-face of the flavin photoadduct (N476L; I489V; V478T/I/L) or by changing the electronic density of residues at the flavin *re*-face (V525R; L558I) (Fig. 1*A*). Importantly, the mutation of these residues did not alter the overall absorption profile of the LOV1+2 protein (*SI Appendix*, Fig. S1*B*) nor did they impair BL-dependent autophosphorylation of full-length phot1 when expressed in insect cells (*SI Appendix*, Fig. S3).

### Photocycle Engineering Modulates the Lifetime of Phot1 Activation *in Planta*.

To examine the impact of photocycle engineering in vivo, we generated transgenic *Arabidopsis* expressing each of the phot1 variants introduced above (Fig. 1*A*) as an N-terminal fusion to GFP. Each variant was expressed in the *phot1phot2* double mutant under the control of the native *PHOT1* promoter. Independent homozygous lines with phot1 protein levels comparable to those in a reference line that expressed wild-type phot1-GFP (p1-WT) (*SI Appendix*, Fig. S4) (38) were then selected. Fast-cycling variants, V478T and I489V, were as effective as p1-WT in restoring leaf expansion (*SI Appendix*, Fig. S5*A*) and leaf positioning (*SI Appendix*, Fig. S5*B*). The complementation of these phenotypes was also observed in the slow photocycle lines N476L, V478I, V525R, and L558I, whereas the V478L line exhibited little complementation of both these phenotypes (*SI Appendix*, Fig. S5).

**Fig. 2.**
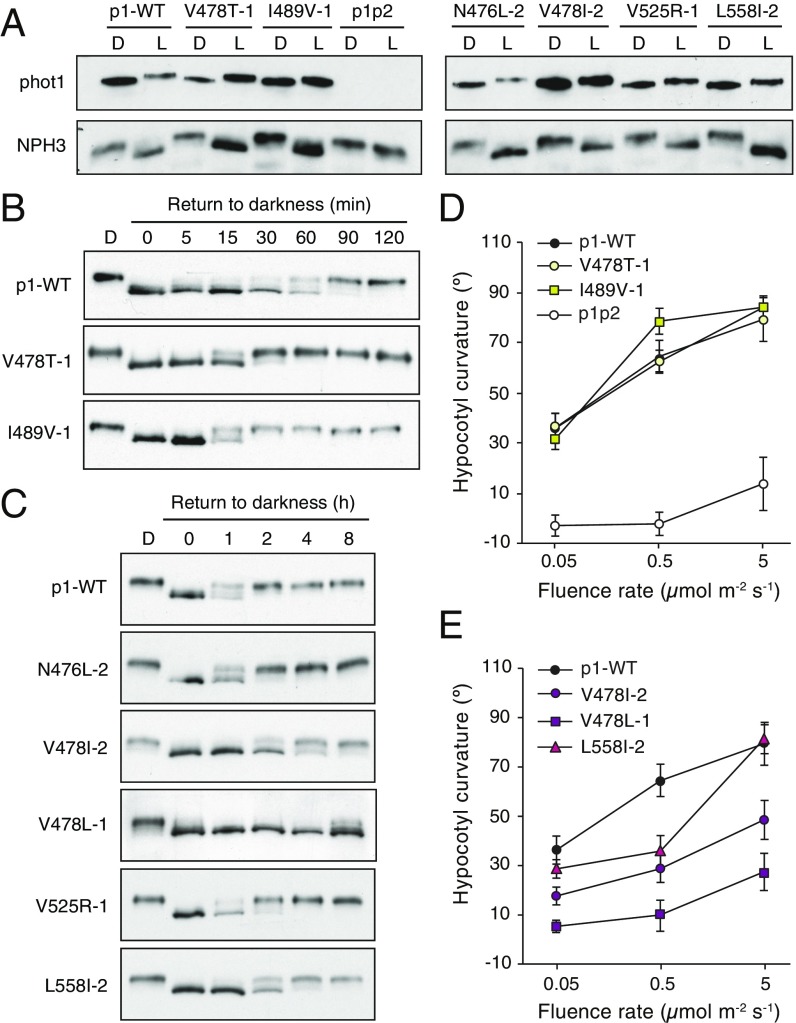
Functionality of the phot1 photocycle mutants in etiolated seedlings. (*A*) Immunoblot analysis of total protein extracts from 3-d-old etiolated *phot1phot2* (p1p2) seedlings expressing phot1-GFP (p1-WT), the fast-cycling variants V478T or I489V, and the slow-cycling variants N476L, V478I, V525R, or L558I. Seedlings were either maintained in darkness (D) or irradiated with 20 μmol m^−2^ s^−1^ of BL for 15 min (L). Blots were probed with an anti-phot1 antibody (*Upper*) or anti-NPH3 antibody (*Lower*). (*B*) NPH3 phosphorylation status in etiolated seedlings expressing p1-WT or the fast-cycling mutants V478T or I489V. Three-day-old seedlings (D) were treated with 20 μmol m^−2^ s^−1^ BL for 15 min then returned to darkness for the times indicated. Protein extracts were isolated for immunoblot analysis with anti-NPH3 antibody. (*C*) NPH3 phosphorylation status in etiolated seedlings expressing p1-WT or the slow-cycling mutants N476L, V478I, V478L, V525R, or L558I. Seedlings were treated and analyzed as in *B*. (*D*) Hypocotyl phototropism in 3-d-old etiolated p1p2 seedlings expressing p1-WT or fast-cycling mutants V478T or I489V. Seedlings were irradiated with unidirectional BL at the fluence rates indicated for 24 h. Curvatures were calculated as the mean ± SE of 20–30 seedlings. (*E*) Hypocotyl phototropism in 3-d-old etiolated p1p2 seedlings expressing p1-WT or the slow-cycling mutants V478I, V478L, or L558I. Seedlings were treated as in *D*.

Following irradiation, the electrophoretic mobility of each of the phot1 variants was reduced, which is indicative of light-dependent autophosphorylation (Fig. 2*A*), including the V487L variant (*SI Appendix*, Fig. S6). Similarly, all of the photocycle variants showed partial internalization from the plasma membrane upon BL excitation (*SI Appendix*, Fig. S7), consistent with their observed light-induced kinase activity in vitro (Fig. 2*A*). We therefore rationalized that this activity should be sufficient to initiate the early events associated with phot1 signaling, such as the rapid dephosphorylation of NONPHOTOTROPIC HYPOCOTYL 3 (NPH3). NPH3 is essential for phototropism (39) and is rapidly dephosphorylated in response to phot1 activation (40). All of the photocycle variants were able to trigger this process (Fig. 2*A* and *SI Appendix*, Fig. S6).

NPH3 is rephosphorylated when seedlings are returned to darkness on a time scale that correlates with the lifetime duration of phot1 activation (41). We therefore investigated whether the kinetics of NPH3 rephosphorylation were altered in each of the photocycle mutant lines. NPH3 rephosphorylation was notably faster in the V478T and I489V lines compared with p1-WT (Fig. 2*B*), whereas it was slower in the V478I, V478L, and L558I lines (Fig. 2*C*). Delayed NPH3 rephosphorylation was particularly striking in the V478L line. No obvious differences were detected between p1-WT and the lines expressing N476L or V525R (Fig. 2*C*), indicating that the successful modulation of adduct decay in vitro does not always correlate with altered photoreceptor activity in vivo. However, the efficacy of NPH3 rephosphorylation as a proxy for monitoring the lifetime duration of phot1 activation *in planta* enabled us to prioritize the fast-cycling variants V478T and I489V and the slow-cycling variant V478L, V478I, and L558I for further study.

### Slow Photocycle Tuning Impairs Phototropism.

Phototropism, in response to a range of BL fluence rates, was first examined to determine whether any of the phot1 photocycle mutants displayed altered sensitivity to BL. Lines that express the fast-cycling variants V478T and I489V were comparable in their response to p1-WT (Fig. 2*D*). By comparison, each of the slow-cycling variants showed a reduced phototropic fluence-rate response relative to p1-WT (Fig. 2*E*). Equivalent phototropism results were obtained using independent lines (*SI Appendix*, Fig. S8). The rephosphorylation of NPH3 is considered to be important for promoting efficient phototropism under continuous irradiation (39, 41). The sustained level of NPH3 dephosphorylation observed in lines expressing the slow-cycling variants V478I, V478L, and L558I (Fig. 2*E*) may therefore correlate with a reduction in phototropic responsiveness. Indeed, mutation of V525 to arginine in phot1, which did not alter NPH3 rephosphorylation (Fig. 2*E*), had no impact on phototropism (*SI Appendix*, Fig. S9). Phototropism was severely impaired in the V478L line (Fig. 2*E*), which showed a marked effect on NPH3 rephosphorylation (Fig. 2*C*). However, the mutation of V478 to leucine, but not to isoleucine, also severely impeded the ability of phot1 to mediate leaf expansion and positioning (*SI Appendix*, Fig. S5) without affecting its kinase activation (*SI Appendix*, Figs. S3, S6, and S7). This indicates that the V478L variant is generally detrimental to phot1 signaling.

Phototropic curvature depends on the formation of a gradient of photoreceptor activation across the hypocotyl to drive differential and directional growth (5). It is possible that a gain in sensitivity associated with the slow photocycle of the V478I and L558I variants could ameliorate the formation or duration of this gradient by saturating photoreceptor across a wider range of fluence rates. We therefore explored the responsiveness of these photocycle mutants for other phot1-mediated responses.

### Photocycle Tuning Alters Phot1 Sensitivity for Chloroplast Accumulation.

**Fig. 3.**
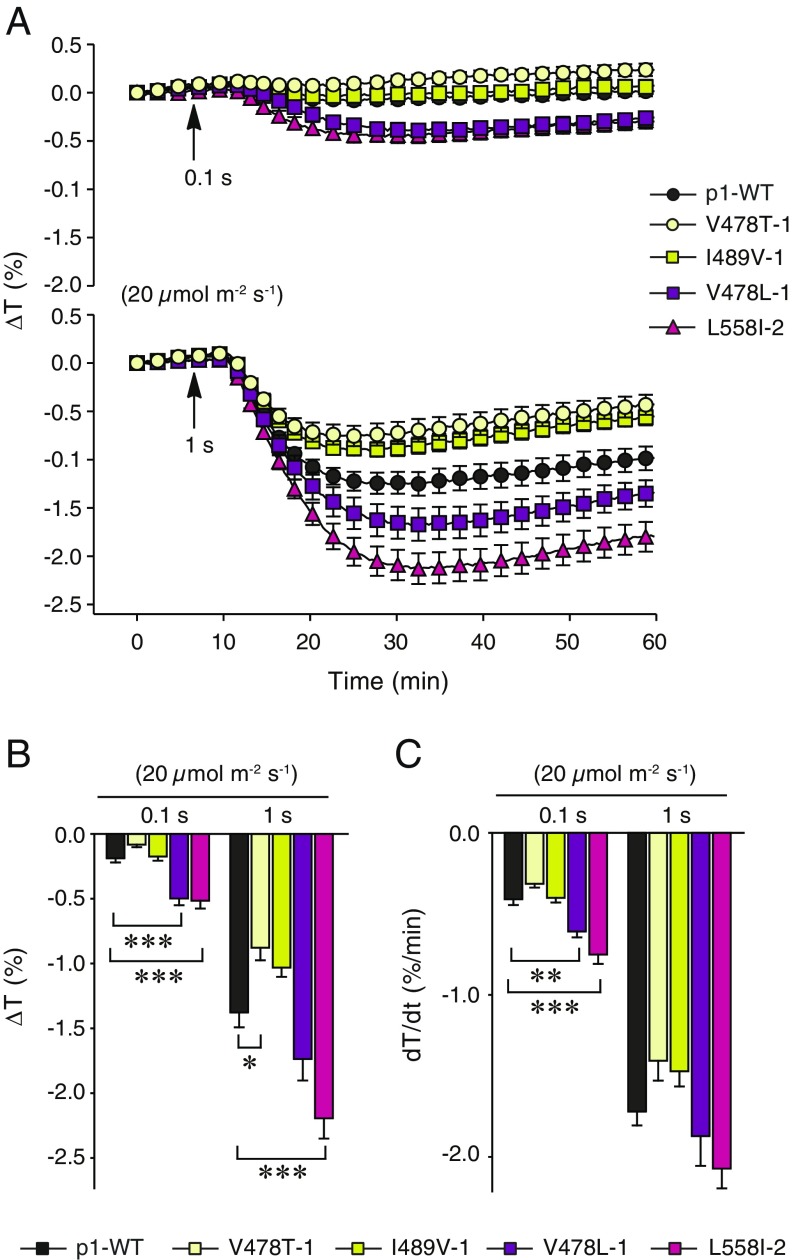
Chloroplast movement in the phot1 photocycle mutants. (*A*) Time course of changes in the leaf transmittance induced by a 0.1- or 1-s pulse of 20 µmol m^−2^ s^−1^ BL. Amplitudes (*B*) and maximal rates (*C*) of transient chloroplast accumulation. Measurements were taken on detached rosette leaves from 4-wk-old plants, grown under 70 µmol m^−2^ s^−1^ WL (10/14 h L/D cycles). Each value is the mean ± SE of 12–14 measurements. Asterisks indicate significant differences between photocycle mutants and the p1-WT line (*P = 0.01–0.05; **P = 0.001–0.01, ***P < 0.001, one-way ANOVA with Dunnett’s posttest, calculated for each light dose separately).

Both continuous and pulses of BL promote chloroplast movements (42). Thus, the light transmittance properties of leaves were examined to acquire kinetic measurements of chloroplast accumulation. A short 0.1- or 1-s pulse of BL (20 µmol m^−2^ s^−1^) was sufficient to induce a chloroplast accumulation response in *Arabidopsis* expressing p1-WT, which was evident by a transient decrease in red light (RL) transmittance through the leaf (Fig. 3*A*). The amplitude of this response was reduced in lines expressing the fast-cycling variants V478T and I489V. By contrast, the responses of the slow-cycling variants, V478L and L558I, were notably higher in magnitude relative to p1-WT (Fig. 3 *A* and *B*). A similar correlation was observed when 0.1- or 1-s pulse durations of 120 µmol^−2^ s^−1^ were applied (*SI Appendix*, Fig. S10 *A* and *B*). Accelerating or slowing down the phot1 photocycle was also found to affect the rate of chloroplast accumulation movement. Slow-cycling variants showed increased response rates with 0.1-s pulse durations, whereas lines harboring fast-cycling variants tended to show delayed responsiveness (Fig. 3*C* and *SI Appendix*, Fig. S10*C*).

The above-mentioned changes in light sensitivity coincides with the effects of these photocycle mutations on photoreceptor performance. At steady-state levels, the photoreceptor pool would be balanced by active and inactive molecules. Increasing the rate of adduct decay would reduce the pool of active photoreceptors, thereby decreasing the plant’s sensitivity to BL. In contrast, limiting adduct decay would increase the population of active photoreceptors and enhance the plant’s sensitivity. Taken together, these findings demonstrate that photocycle tuning can alter phot1 responsiveness to BL pulses *in planta*. Moreover, slowing the photocycle can lead to phot1 becoming more sensitive to BL. Substitution of V478 to leucine (Fig. 3) or isoleucine (*SI Appendix*, Fig. S11) enhanced chloroplast accumulation. However, the V478L variant displayed impairments for phototropism (Fig. 2*E*), leaf expansion, and leaf positioning (*SI Appendix*, Fig. S5). Thus, this mutation appears to adversely affect NPH3-dependent processes (39), which might correlate with its impact on NPH3 dephosphorylation (Fig. 3*C*) because NPH3 dephosphorylation has been linked to a cessation of photoreceptor signaling (41).

### Slow Photocycle Tuning Increases Phot2 Responsiveness.

Phot1 is known to exhibit a slower photocycle than phot2 (9, 34), which is proposed to contribute to the greater sensitivity of phot1 to BL in comparison with phot2 (9, 43–45). We therefore examined whether slow photocycle tuning could enhance phot2’s responsiveness. We did so by targeting V392 in phot2 for mutagenesis; this residue is equivalent to V478 in phot1. The mutation of V392 to leucine slowed the rate of phot2 adduct decay in vitro (*SI Appendix*, Fig. S12), as it did for phot1 (Fig. 1*C*). Thus, independent homozygous lines that express comparable levels of WT phot2-GFP (p2-WT) and the V392L variant were generated in the *phot1phot2* double mutant for further analysis (*SI Appendix*, Fig. S13).

**Fig. 4.**
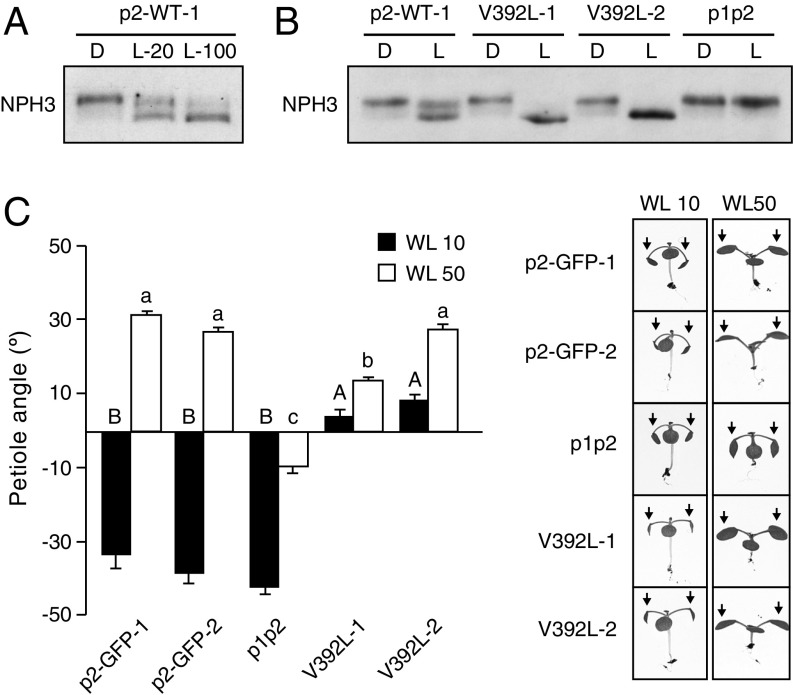
Functionality of phot2 and its V392L variant in *Arabidopsis*. (*A*) Immunoblot analysis of total protein extracts from 3-d-old etiolated *phot1phot2* (p1p2) seedlings expressing phot2-GFP (p2-WT). Seedlings were either maintained in darkness (D) or irradiated with either 20 μmol m^−2^ s^−1^ BL for 15 min (L-20) or 100 μmol m^−2^ s^−1^ of BL for 60 min (L-100) and probed with an anti-NPH3 antibody. (*B*) NPH3 phosphorylation status in etiolated seedlings expressing p2-WT or the slow-cycling variant V392L in response 20 μmol m^−2^ s^−1^ BL for 15 min. (*C*) Petiole positioning responses in lines expressing p2-WT or the slow-cycling mutant V392L. Two independent homozygous lines were examined in each case. Plants were grown under 80 μmol m^−2^ s^−1^ WL (16/8 h L/D cycles) for 7 d before being transferred to 10 μmol m^−2^ s^−1^ (WL 10) or 50 μmol m^−2^ s^−1^ (WL 50) WL (16/8 h L/D cycles) for 5 d. Petiole angle from the horizontal was measured for the first true leaves. Each value is the mean ± SE of 10 seedlings. Means that do not share a letter are significantly different (*P* < 0.001, one-way ANOVA with Games–Howell posttest). Representative images for each genotype are shown on the right for each WL condition.

The abundance of phot2 is very low in etiolated *Arabidopsis* seedlings (4) and it is not able to trigger light-induced dephosphorylation of NPH3 (40). However, when phot2 is expressed from the *PHOT1* promoter, it is produced in sufficient levels in etiolated seedlings to partly stimulate NPH3 dephosphorylation in response to BL exposure (Fig. 4*A*). This response was more pronounced at higher light intensities, indicating that it depends on the sensitivity of phot2. The V392L variant was more effective at mediating this response compared with p2-WT at lower light intensities (Fig. 4*B*), which is in accordance with this mutation increasing phot2 performance. The enhanced responsiveness of the V392L line was also observed by increased leaf positioning (Fig. 4*C*) and expansion relative to p2-WT (*SI Appendix*, Fig. S14). However, the substitution of V392 to leucine in phot2 had a detrimental effect on phototropism (*SI Appendix*, Fig. S15), as was found for the equivalent mutation in phot1 (Fig. 2*E*). These findings once again highlight that slow photocycle tuning impairs phototropism. Thus, we next examined the impact of the V392L mutation on phot2-induced chloroplast movement.

### Photocycle Tuning Alters the Light Intensity Threshold for Chloroplast Avoidance Movement.

**Fig. 5.**
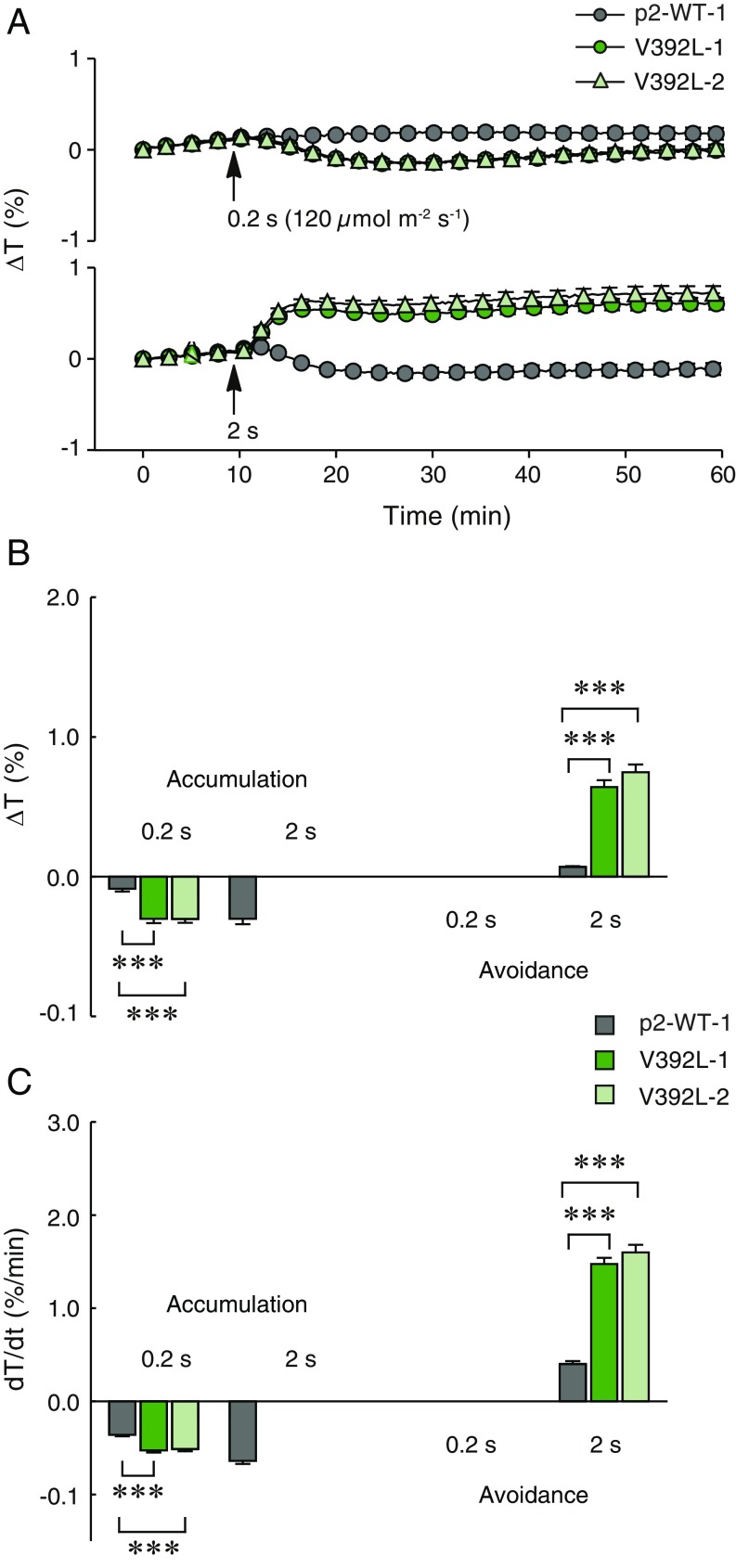
Chloroplast responses in lines that express the V392L variant of phot2. Chloroplast movement to a 0.2- or 2-s-long pulse of 120 µmol m^−2^ s^−1^ BL was measured in detached rosette leaves of 4-wk-old plants that express phot2-WT and in two independent lines that express the V392L variant. (*A*) Time course of changes in the leaf transmittance, (*B*) amplitudes and (*C*) maximal rates of chloroplast accumulation and avoidance induced by BL pulses. Each value in *A–C* is the mean ± SE of 26 measurements. Asterisks indicate significant differences between photocycle mutants and the p2-WT line ***P < 0.001, one-way ANOVA with Dunnett’s posttest, calculated for each light dose separately).

The function of phot2 in regulating chloroplast movement is different from that of phot1. Phot2, like phot1 (46), can mediate chloroplast accumulation movement at low light intensities. However, phot2, unlike phot1, switches its function at higher light intensities to control chloroplast avoidance movement. A short 0.2-s pulse of BL (120 µmol m^−2^ s^−1^) elicited a weak chloroplast accumulation response in lines expressing p2-WT (Fig. 5 *A* and *B*). Longer pulse durations of 2 (Fig. 5 *A* and *B*) and 20 s (*SI Appendix*, Fig. S16) triggered a biphasic response in which leaf transmittance increased (avoidance movement) and then subsequently decreased (accumulation movement) over time, as reported previously (42). Lines harboring the V392L variant showed a greater and more sustained responsiveness to BL compared with p2-WT (Fig. 5*A* and *SI Appendix*, Fig. S16). A biphasic response was not observed for the V392L lines as the return of chloroplasts to their dark positioning after avoidance movement was much slower compared with p2-WT. The V392L lines also showed increased response rates for chloroplast avoidance and accumulation movement at shorter pulse durations of 0.2 and 2 s (Fig. 5*C*). These data, combined with those obtained for leaf positioning (Fig. 4*C*) and leaf expansion (*SI Appendix*, Fig. S14), demonstrate that the V392L mutation enhances the sensitivity of phot2 for multiple responses.

The switch between chloroplast accumulation and avoidance movement occurs at a specific light intensity. However, how this switch in chloroplast movement is regulated remains unknown. The transition between chloroplast accumulation and avoidance movement was readily observed in our analysis (Fig. 5*C*). Notably, the light-intensity threshold for mediating chloroplast avoidance movement was lower in the lines expressing V392L, consistent with this line’s enhanced sensitivity to BL. These findings therefore demonstrate that the light intensity for the transition to chloroplast avoidance movement is governed by the photocycle of phot2. A decrease in temperature can also promote chloroplast avoidance movement in *Arabidopsis* at lower light intensities (47, 48). Our results are in agreement with the proposal that temperature can modify the action of phot2 by altering its photocycle (47). Prolonging photoproduct lifetime by either reducing the temperature or by photocycle engineering, as shown here, would increase phot2 sensitivity and shift the light threshold for chloroplast avoidance movement to lower light intensities.

### Photocycle Engineering of Phot1 and Phot2 Increases Plant Biomass Production.

Efficient photosynthetic capacity and plant growth depends on the accumulation of chloroplasts at the periclinal walls of the mesophyll (49). We therefore examined whether the enhanced chloroplast positioning recorded in the slow-cycling variants of phot1 (Fig. 3) and phot2 (Fig. 4) could increase growth low-light environments.

**Fig. 6.**
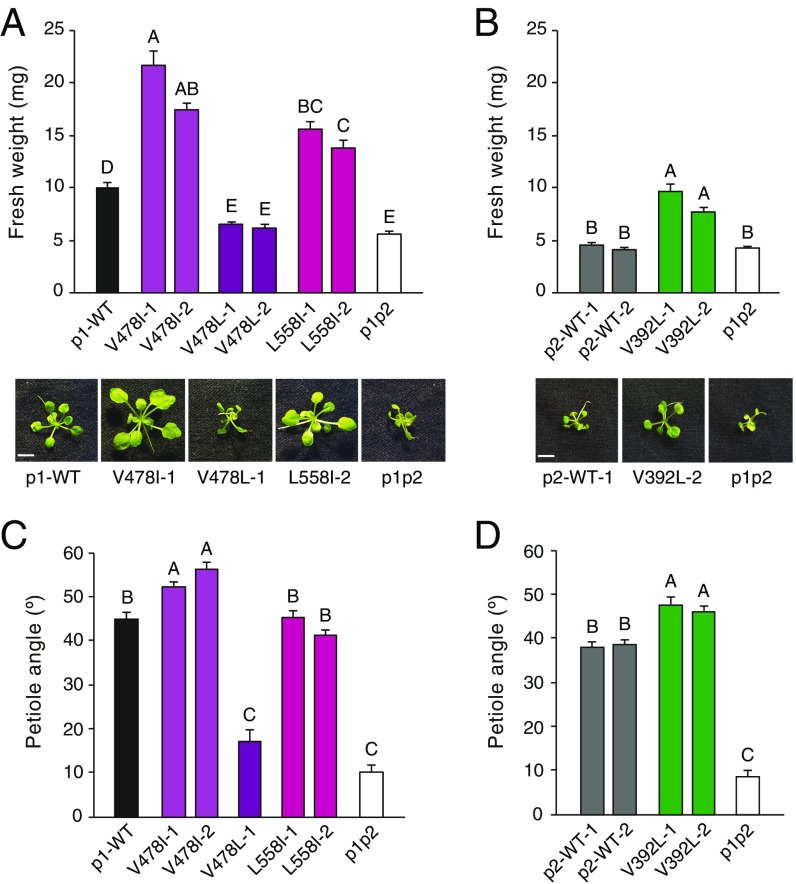
Slow photocycle engineering of phot1 and phot2 increases responsiveness and plant biomass production at low light intensities. (*A*) Growth and biomass production in *phot1phot2* (p1p2) plants that express phot1-GFP (p1-WT) or a slow-cycling mutants V478I, V478L, or L558I. Plants were grown in 25 μmol m^−2^ s^−1^ of RL + 0.1 μmol m^−2^ s^−1^ BL for 4 wk before above ground tissue was harvested for fresh weight determination. Each value is the mean ± SE of 20 seedlings. Means that do not share a letter are significantly different (*P* < 0.001, one-way ANOVA with Games–Howell posttest). (*B*) Growth and biomass production in *phot1phot2* (p1p2) plants that express phot2-GFP (p2-WT) or the slow-cycling variant V392L, as determined in *A*. (Scale bars, 5 mm.) Representative images for each genotype are shown beneath each graph. (*C*) Petiole positioning responses in the phot1 transgenic lines. Plants were grown under 80 μmol m^−2^ s^−1^ WL (16/8 h L/D cycles) for 7 d before being transferred to 25 μmol m^−2^ s^−1^ of RL + 0.1 μmol m^−2^ s^−1^ BL for 5 d. Each value is the mean ± SE of 20–25 seedlings. Means that do not share a letter are significantly different (*P* < 0.001, one-way ANOVA with Games–Howell posttest). (*D*) Petiole positioning responses in the phot2 transgenic lines as determined in *C*.

Plants were grown under 25 µmol m^−2^ s^−1^ RL, which is limiting for photosynthesis (50), and were supplemented with weak BL (0.1 µmol m^−2^ s^−1^) to stimulate basal phototropin activation. Lines that express either the V478I or L558I variant of phot1 showed twofold increased biomass production under these light conditions compared with p1-WT (Fig. 6*A*). Immunoblot analysis confirmed that these functional enhancements did not result from a greater abundance of phot1 (*SI Appendix*, Fig. S17*A*). The phenotype of the V478L line, however, was indistinguishable from that of the *phot1phot2* double mutant. Although the V478L variant showed enhanced responsiveness for chloroplast accumulation (Fig. 3), it displayed impaired functionality in terms of leaf expansion and leaf positioning (*SI Appendix*, Fig. S5). We therefore concluded that the increased biomass detected in the V478I and L558I variants was not caused solely by enhanced chloroplast accumulation. Instead, these lines must also depend on efficient leaf positioning and leaf expansion. Leaf positioning in the L558I line was comparable to that of p1-WT in response to low RL+BL, whereas the V478I variant exhibited greater responsiveness relative to p1-WT (Fig. 6*C*). The greater impact of the V478I mutation on plant growth correlates with its ability to enhance both leaf positioning and chloroplast accumulation.

Growth enhancements were also evident in the lines expressing the V392L variant of phot2 grown under equivalent light conditions (Fig. 6*B*). Again, these enhancements were not due to increased phot2 protein levels (*SI Appendix*, Fig. S17*B*). The V392L variant also displayed increased responsiveness in terms of leaf positioning under these light conditions (Fig. 6*D*). We therefore concluded that increased biomass production in the slow-cycling variants under light-limiting conditions stems from their ability to coordinate enhancements in one or more light-capturing process.

Taken together, our findings demonstrate the feasibility of fine-tuning the phototropin photocycle to confer improved protein functionality and responsiveness in *Arabidopsis*. We anticipate that this approach will contribute to coordinating stepwise enhancements in photosynthesis to increase yield by growing crops more efficiently, particularly in low-light environments, which could be relevant to agricultural practices, such as intercropping. Phototropins also enhance photosynthetic capacity by controlling stomatal opening (51). Thus, the synthetic biology approach used here might also offer new opportunities to modulate stomatal responsiveness and to improve water use efficiency without impeding carbon fixation.

*Arabidopsis* mutants that lack phot2 have enhanced leaf photosynthesis as a consequence of no longer being able to mediate chloroplast avoidance movement (52). However, such a strategy for increasing plant biomass comes at a cost, as plants lacking such movements are susceptible to photodamage (53). The work outlined here thus establishes a more robust approach for engineering photosensory modules that aim to further optimize leaf photosynthesis. Accelerating the photocycle of phot2 would increase the light intensity threshold for chloroplast avoidance movement. This, in combination with the slow photocycle tuning of phot1, would synchronize their actions more effectively over a wider range of light conditions without causing detrimental high light stress. Indeed, the transitional light intensity for chloroplast positioning responses differs widely among plant species (54). Harnessing this natural diversity to engineer additional changes in phot2 sensitivity, and the natural variation available in *Arabidopsis*, should also prove to be valuable for identifying further variants that could improve photoreceptor performance.

## Methods

### Plant Material and Growth.

Wild-type *Arabidopsis* (*gl-1*, ecotype Columbia) and the *phot1-5phot2-1* double mutant were described previously (46), as was the phot1-GFP transgenic line (38). Unless otherwise noted, seeds were sown on soil and stratified for 2 d at 4 °C before being transferred to the light condition of interest. For fresh weight experiments, plants were grown for 4 wk in a LED growth cabinet (MD1400, CEC Technologies) under a 16/8 h 22/18 °C light/dark cycle. The statistical significance of the effects of plant lines on fresh weight was assessed by one-way ANOVA followed by Games–Howell posttest (Minitab Statistical Software). Fluence rates for all light sources were measured with a Li-250A and quantum sensor (LI-COR).

### Protein Purification and Spectral Analysis.

*Arabidopsis* phot1 LOV1+2 (amino acid residues 180–628) was expressed using the pCAL-n-EK vector (Agilent) to create a N-terminal calmodulin-binding peptide fusion and purified by calmodulin affinity chromatography, as described previously (10). Amino acid substitutions were introduced using the QuikChange site-directed mutagenesis kit (Agilent). Absorption spectra were measured using a Shimadzu MultiSpec-1501 diode array spectrophotometer at room temperature (26). A protein concentration of 1.8 mg mL^−1^ was used for measurements and the optical path length was 0.5 cm. Protein concentration was calculated from the absorbance at 450 nm, according to Beer’s Law, using 12,500 M^−1^ cm^−1^ as the molecular absorptivity of LOV domains (55).

### Insect Cell Expression and in Vitro Kinase Assays.

Recombinant baculovirus was generated using the Bacmagic transfection kit (Merck Millipore) in accordance with the supplier’s instructions. Mutation and expression of recombinant phot1 was performed as previously described (26). Kinase assays were performed as previously described (26). Protein extract (10 μg) was either mock irradiated under RL or treated for 10 s with white light (WL) at a total fluence of 10,000 μmol m^−2^. Reactions were performed for 2 min at room temperature and terminated by the addition of SDS sample buffer.

### Transformation of *Arabidopsis*.

Amino acid substitutions were introduced into pBlueScript SK+ or pAcHLT-A vectors containing the full-length coding sequence of *PHOT1* or *PHOT2* using the QuikChange site-directed mutagenesis kit (Agilent) and verified by DNA sequencing. Plant transformation vectors were generated by replacing the coding sequence of *PHOT1* in the *PHOT1*::*PHOT1-GFP* pEZR(K)-LN binary vector (56) with the coding sequences containing the photocycle mutations using Gibson Assembly (New England Biolabs). The *phot1-5phot2-1* mutant was transformed with *Agrobacterium tumefaciens* strain GV3101, as previously described (57). Based on the segregation of kanamycin resistance, independent homozygous T3 lines were selected for analysis.

### Immunoblot Analysis.

Total proteins were extracted from 3-d-old etiolated seedlings by directly grinding 50 seedlings in 100 µL of 2× SDS sample buffer under red safe light illumination. Light-grown tissue was frozen and ground to a fine powder in liquid nitrogen before 150 mg of tissue was mixed with 100 µL of 2× SDS sample buffer. Proteins were transferred onto nitrocellulose or polyvinylidene fluoride membrane and detected with anti-phot1 or anti-phot2 polyclonal antibodies (8), anti-NPH3 polyclonal antibody (58) and anti-UGPase polyclonal antibody (Agrisera). Blots were developed with horseradish peroxidase-linked secondary antibodies (Promega) and Pierce ECL Plus Western Blotting Substrate (Thermo Fisher Scientific).

### Phototropism.

Three-day-old etiolated seedlings were grown vertically on half strength MS (Murashige and Skoog) agar plates before being exposured to a unilateral BL stimulus at the indicated fluence rate for 24 h. Images of the seedlings were captured at the end of the light treatment and hypocotyl curvature measured using Fiji software (59).

### Leaf Positioning and Leaf Expansion.

Measurements of leaf positioning and expansion were performed as previously described (60). For leaf positioning, seedlings were grown on soil under 80 µmol m^−2^ s^−1^ of WL in 16/8 h light/dark cycle for 7–9 d before being transferred to the light conditions indicated for 5 d. Seedlings were transferred to agar plates, imaged and petiole angles from the horizontal measured using Fiji software. Leaf expansion was measured on 4-wk-old, soil grown plants. Leaf areas were measured before and after uncurling using Fiji software, and the ratio of the curled to uncurled area was designated as the leaf expansion index. The statistical significance of the effects of plant lines on leaf positioning and leaf expansion parameters was assessed by one-way ANOVA followed by Games–Howell posttest (Minitab Statistical Software).

### Chloroplast Movement.

For chloroplast-movement measurements, plants were sown in Jiffy-7 pots (Jiffy Products International), placed at 4 °C for 2 d and grown in a growth chamber (Sanyo MLR 350H) at 23 °C, 80% relative humidity, with a photoperiod of 10-h light and 14-h dark. WL of 70 µmol m^−2^ s^−1^ was supplied by fluorescent tubes. Four-to five-week-old plants were used to quantify chloroplast movements using a photometric method described previously (42).

Weak RL (0.3 µmol m^−2^ s^−1^, 660 nm, modulated at a frequency of 800 Hz) was used for recording changes in leaf transmittance caused by chloroplast relocations. Actinic BL was supplied by LED Luxeon Royal Blue LXHL-FR5C (Philips Lumiled Lighting Comp, 455 nm). Measurements were recorded on detached rosette leaves of plants, which were dark-adapted for at least 12 h. The initial transmittance level was recorded for 10 min. For phot1 photocycle mutants, 0.1- or 1-s-long pulses of 20 µmol m^−2^ s^−1^ or 120 µmol m^−2^ s^−1^ BL were applied. For phot2 photocycle mutants, 0.2-, 2-, or 20-s-long pulses of 120 µmol m^−2^ s^−1^ BL were applied. Pulse-induced changes in transmittance were recorded for 50 min. Amplitudes of transmittance changes were calculated relative to the dark transmittance level. The maximal rate of transmittance change was calculated as the derivative of the photometric curve, using a Savitzky–Golay filter, with the window width set to 1 min, using a custom-written package in Mathematica 9.0 (Wolfram Research). The statistical significance of the effects of plant lines on chloroplast movement parameters was assessed with ANOVA. Dunnett’s test was used for pairwise comparisons between phot-GFP expressing plants, treated as a control, and photocycle mutants. Statistical calculations were performed with the R software. Dunnett’s test was applied separately for each light treatment.

### Confocal Microscopy.

The localization of GFP-tagged phot1 in hypocotyl cells of 3-d-old etiolated seedlings was visualized with a laser-scanning confocal microscope (Leica SP8) using a HC PL APO 40×/1.30 oil-immersion objective. The 488-nm excitation line was used and GFP fluorescence collected between 500 and 530 nm. Maximum projection images were constructed from *z*-stacks using Fiji software (59).

## Supplementary Material

Supplementary File
