# Supplementary material for: Engineering the phototropin photocycle improves photoreceptor performance and plant biomass production
Source: Proc Natl Acad Sci U S A. 2019 Jun 3;116(25):12550–7. doi: 10.1073/pnas.1902915116 (PMC6589663; doi:10.1073/pnas.1902915116)
Supplement: Supplementary File [file pnas.1902915116.sapp.pdf]

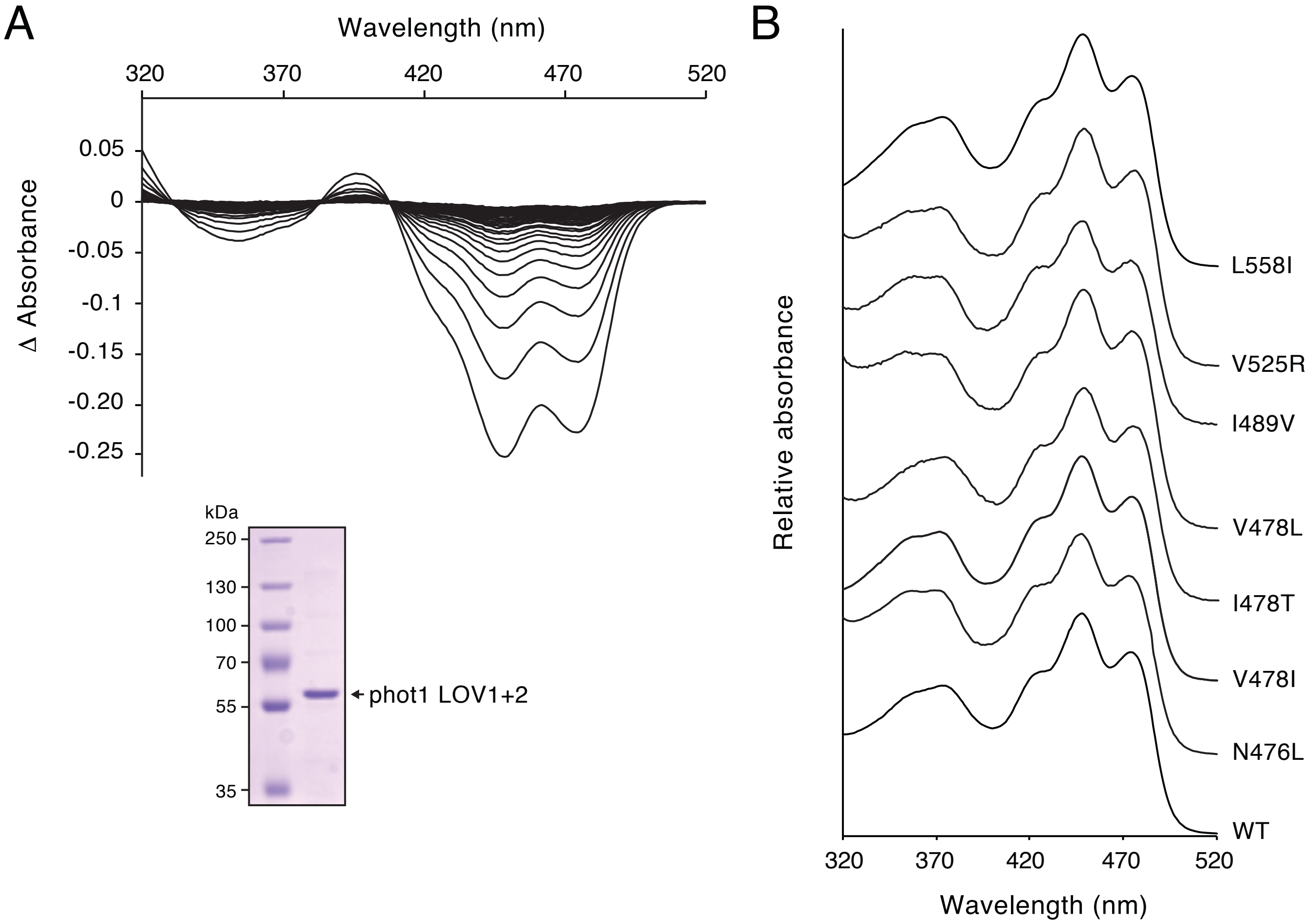

Fig. S1. Spectral properties of the fast- and slow-cycling variants of phot1 LOV1+2.
(*A*) Light-minus-dark absorption difference spectra for phot1 LOV1+2 monitoring adduct recovery back to the initial dark state following photoexcitation. Spectra were recorded every 30 s. Coomassie-stained SDS-PAGE of phot1 LOV1+2 (10 µg) purified from *E. coli* is shown below. (*B*) Absorption spectra for phot1 LOV1+2 (WT) and fast/slow cycling variants. Spectra are offset for clarity.

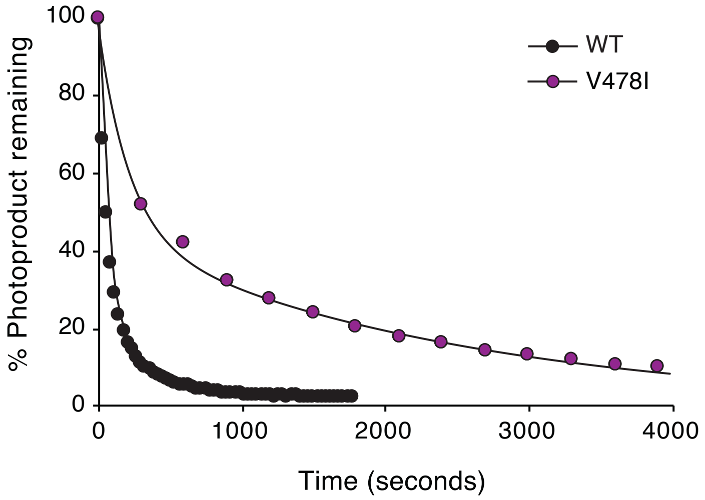

Fig. S2. Adduct decay kinetics for the slow-cycling phot1 LOV1+2 variant V478I.
Purified phot1 LOV1+2 protein was irradiated with a WL camera strobe flash and adduct decay was recorded at an absorbance of 450 nm. Decay is expressed as percentage of photoproduct remaining and was calculated from the ratio of absorbance at 450 nm obtained immediately after the light treatment to that obtained for subsequent sampling in darkness. Decay was fitted to two exponentials with a half-life of 147 and 1733 s. Adduct decay for wild-type phot1 LOV1+2 (WT) is included for reference.

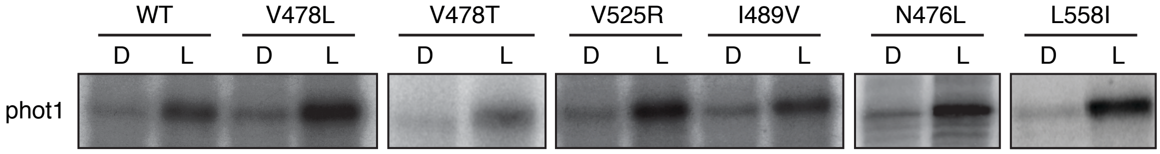

Fig. S3. Autophosphorylation activity of fast- and slow-cycling phot1 variants in insect cells. Autoradiographs showing light-dependent autophosphorylation of wild-type (WT) phot1, as well as N476L, V478L, V478T, I489V, V525R and L558I variants in protein extracts isolated from insect cells. Protein extracts were prepared under dim RL and given a mock irradiation (D) or irradiated with white light (L) prior to the addition of radiolabeled ATP.

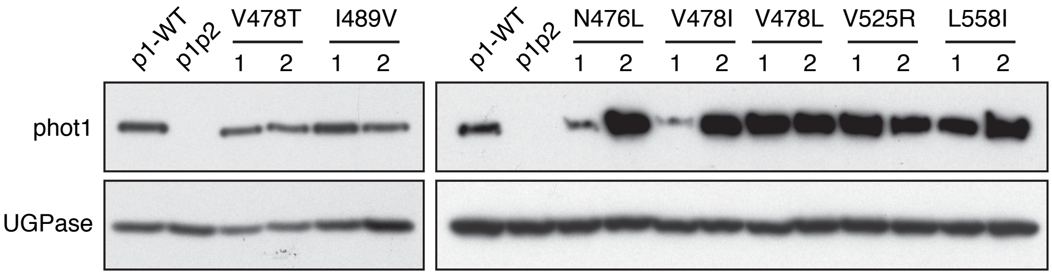

Fig. S4. Expression of phot1 photocycle mutants in the *phot1phot2* mutant of *Arabidopsis*. Immunoblot analysis of total protein extracts isolated from 3-day-old etiolated *phot1phot2* (p1p2) seedlings expressing phot1-GFP (p1-WT), the fast-cycling variants V478T or I489V and the slow-cycling variants N476L, V478I, V478L, V525R or L558I. Two independent homozygous lines (1-2) were examined for each of the photocycle mutants. Protein extracts were probed with an anti-phot1 antibody (upper panel) or an antibody raised against UDP-glucose pyrophosphorylase (UGPase) as a loading control (lower panel).

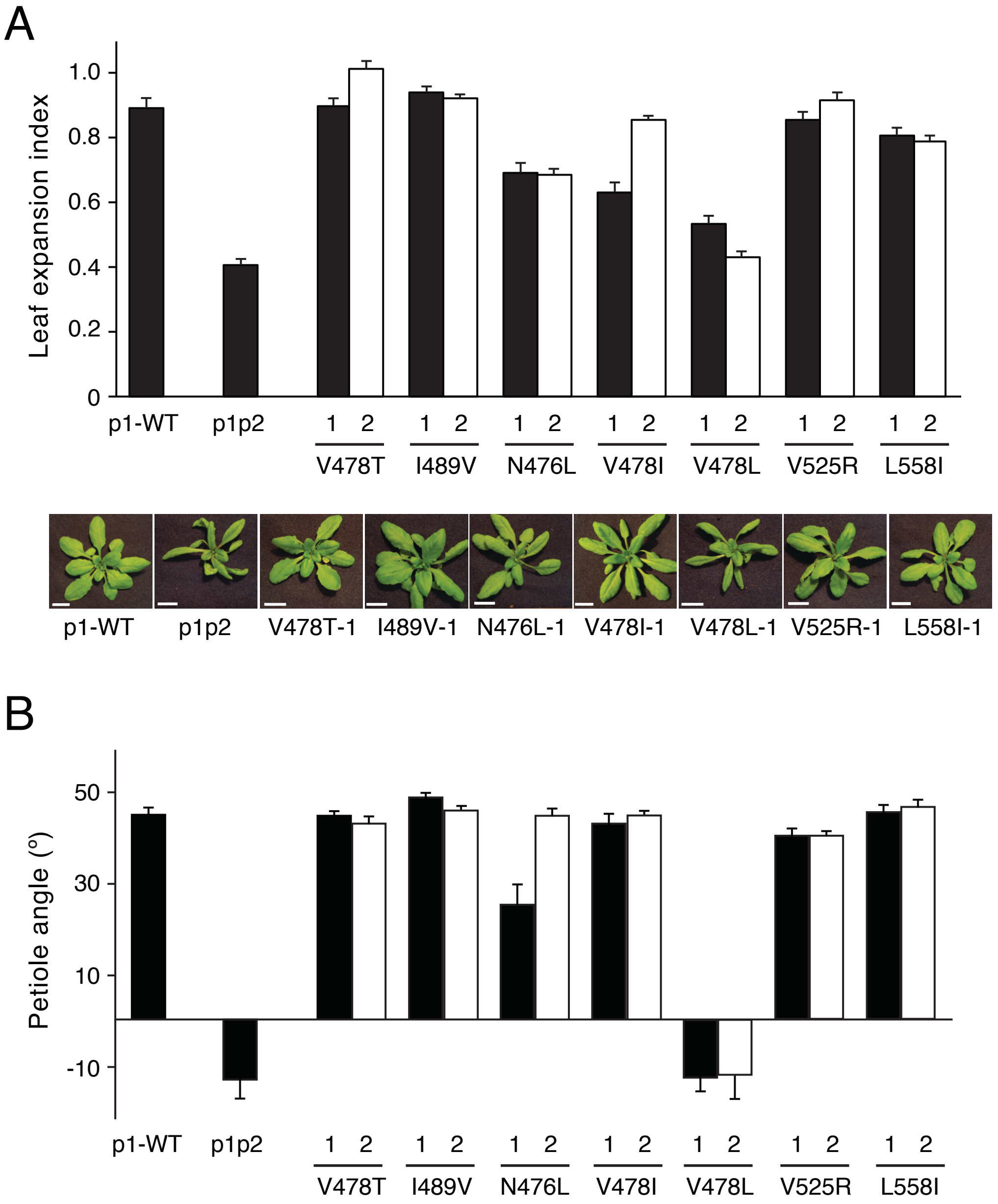

Fig. S5. Leaf expansion and petiole positionig phenotypes in *Arabidopsis* expressing the phot1 photocycle mutants.

Two independent homozygous lines (1-2) were examined for each of the photocycle mutants. (*A*) Leaf expansion responses in *phot1phot2* (p1p2) plants that express phot1-GFP (p1-WT) or the phot1 photocycle mutants indicated. Plants were grown for 4 weeks under 100 µmol m^{-2} s^{-1} white light (16/8 h L/D cycles). The leaf expansion index of the 5th rosette leaf was expressed as the ratio of the leaf area before and after artificial uncurling. Each value is the mean ± S.E. of 12 leaves. Representative images for each genotype are shown below. Bars = 1 cm. (*B*) Petiole positioning responses for phot1 photocycle mutants. Plants were grown under 80 µmol m^{-2} s^{-1} white light (16/8 h L/D cycles) for 7 days and then transferred to 10 µmol m^{-2} s^{-1} white light (16/8 h L/D cycles) for 5 days. Petiole angle from the horizontal was measured for the first true leaves. Each value is the mean ± S.E. of 20-24 seedlings.

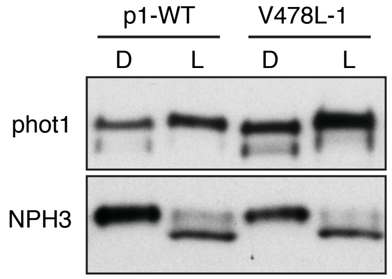

Fig. S6. Activity of the slow photocycle phot1 mutant V478L in etiolated seedlings. Immunoblot analysis of total protein extracts from 3-day-old etiolated *phot1phot2* seedlings expressing phot1-GFP (p1-WT) or V478L. Seedlings were either maintained in darkness (D) or irradiated with 20 μmol m^{-2} s^{-1} of BL for 15 min (L) and probed with anti-phot1 antibody (upper panel) or anti-NPH3 antibody (lower panel).

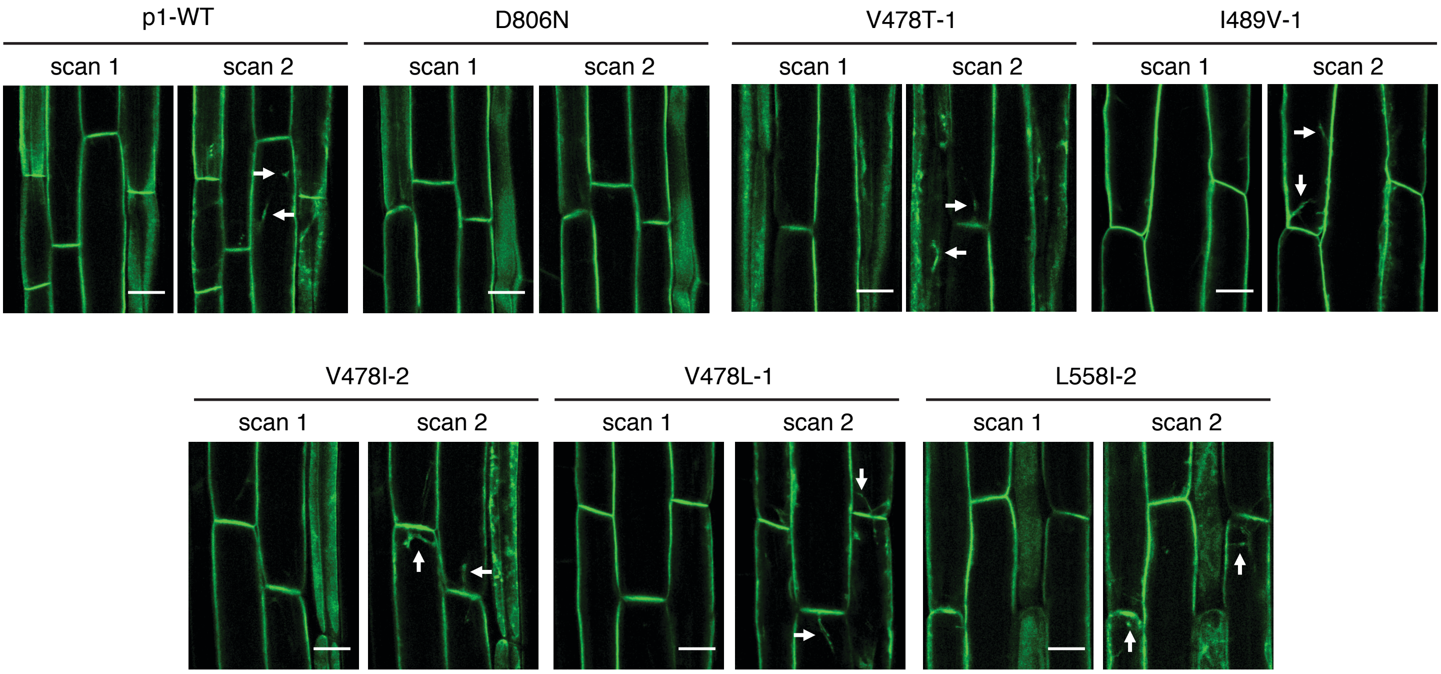

Fig. S7. Blue light-induced changes in the subcellular localization of the phot1 photocycle mutants. Composite maximum projection images of hypocotyl cells from 3-day-old etiolated seedlings scanned immediately (scan 1) and after a 10-min interval (scan 2) with darkness between each scan. Each scan took 120 s to complete. GFP is shown in green. *Arabidopsis* expressing phot1-GFP (p1-WT) and the kinase inactive mutant D806N were included as positive and negative controls, respectively. White arrows indicate phot1-GFP internalization. Bars = 20 μm.

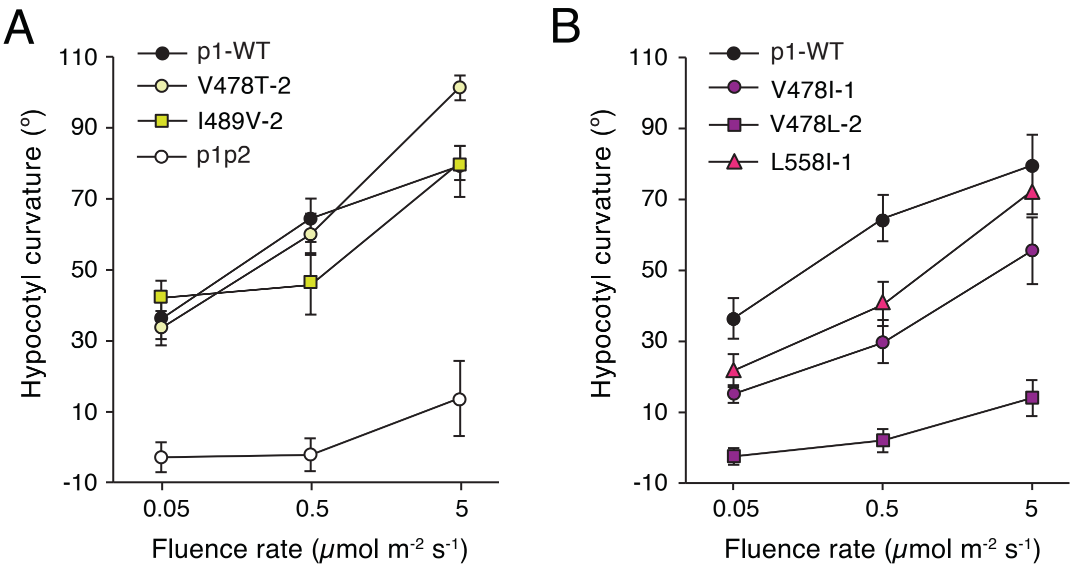

Fig. S8. Phototropic responsiveness of the phot1 photocycle mutants in etiolated seedlings. Hypocotyl phototropism in 3-day-old etiolated *phot1phot2* (p1p2) seedlings that express phot1-GFP (p1-WT), the fast-cycling mutants V478T or I489V (*A*) or the slow-cycling mutants V478I, V478L or L558I (*B*). Seedlings were irradiated with unidirectional BL at the fluence rates indicated for 24 h. Curvatures were calculated as the mean ± S.E. of 20-30 seedlings.

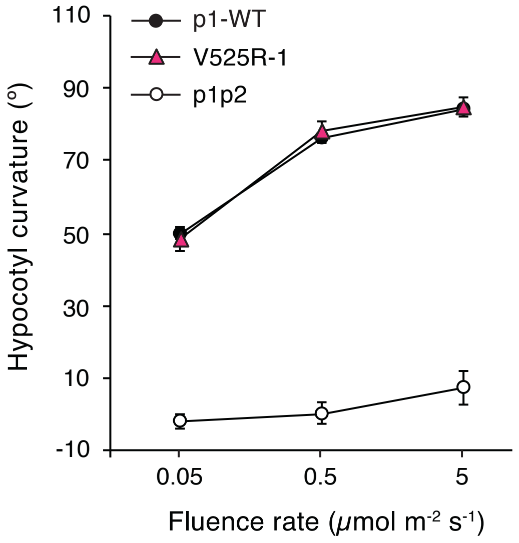

Fig. S9. Phototropic responsiveness of the phot1 V525R mutant in etiolated seedlings. Hypocotyl phototropism in 3-day-old etiolated *phot1phot2* (p1p2) seedlings that express phot1-GFP (p1-WT) or the V525R-1 mutant. Seedlings were irradiated with unidirectional BL at the fluence rates indicated for 24 h. Curvatures were calculated as the mean ± S.E. of 20-30 seedlings.

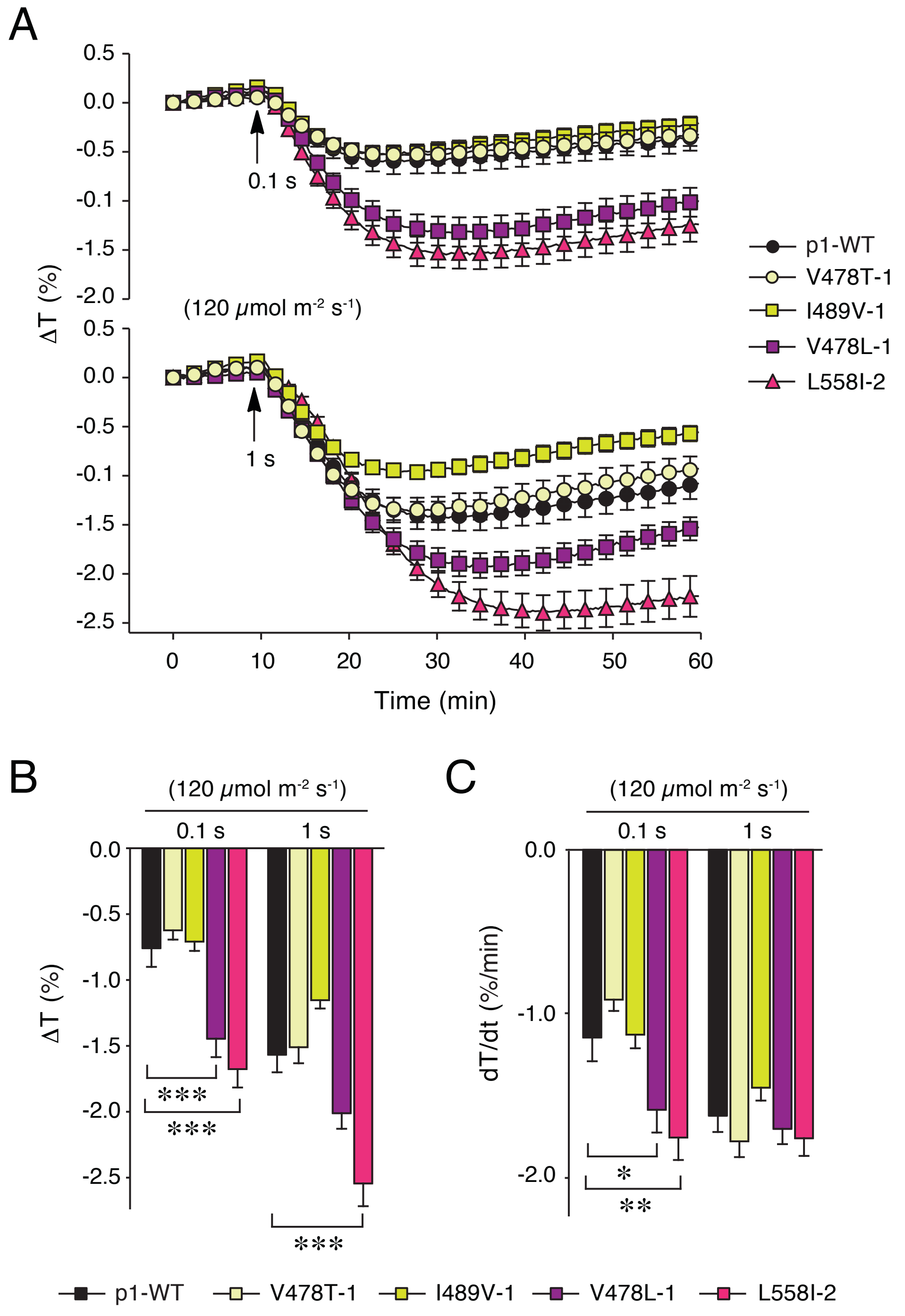

Fig. S10. Chloroplast movement in the phot1 photocycle mutants.
(*A*) Time course of changes in the leaf transmittance induced by a 0.1 or 1 s pulse of 120 μmol m^{-2} s^{-1} BL. Amplitudes (*B*) and maximal rates (*C*) of transient chloroplast accumulation. Measurements were taken on detached rosette leaves from 4-week-old plants, grown under 70 μmol m^{-2} s^{-1} white light (10/14 h L/D cycles). Each value is the mean ± S.E. of 12–14 measurements. Asterisks indicate significant differences between photocycle mutants and the p1-WT line (*P=0.01–0.05; **P=0.001–0.01, ***P<0.001, one-way analysis of variance with Dunnett's post test, calculated for each light dose separately).

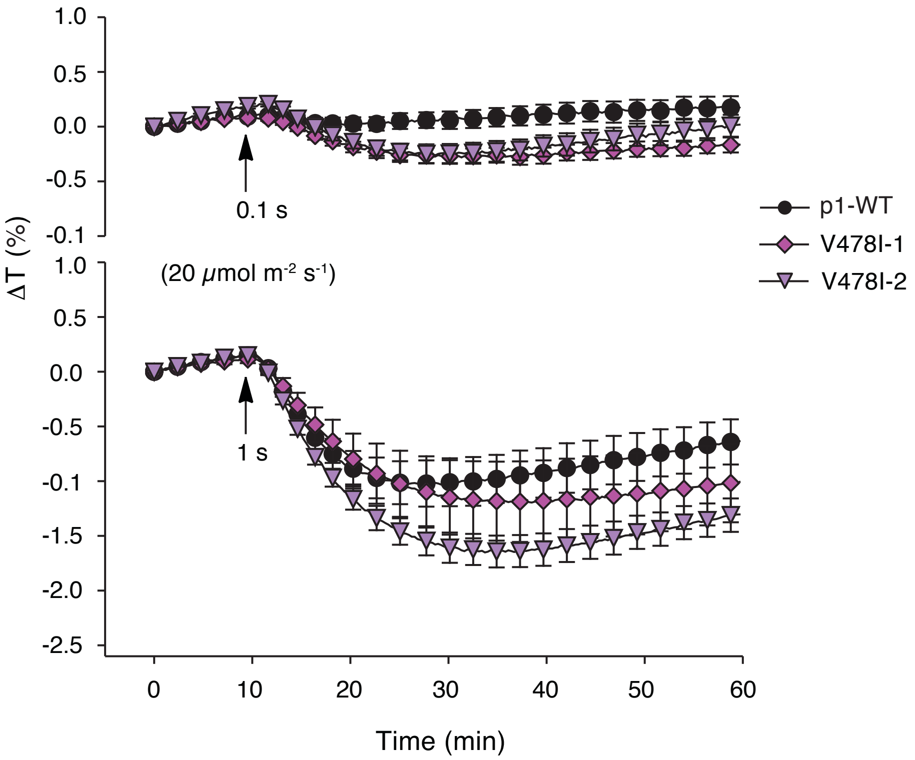

Fig. S11. Chloroplast movement in lines that express the V478I variant of phot1.
Time course of changes in the leaf transmittance induced by a 0.1 or 1 s pulse of 20 μmol m^{-2} s^{-1} BL. Measurements were taken on detached rosette leaves from 4-week-old plants, grown under 70 μmol m^{-2} s^{-1} white light (10/14 h L/D cycles). Each value is the mean ± S.E. of 8–10 measurements.

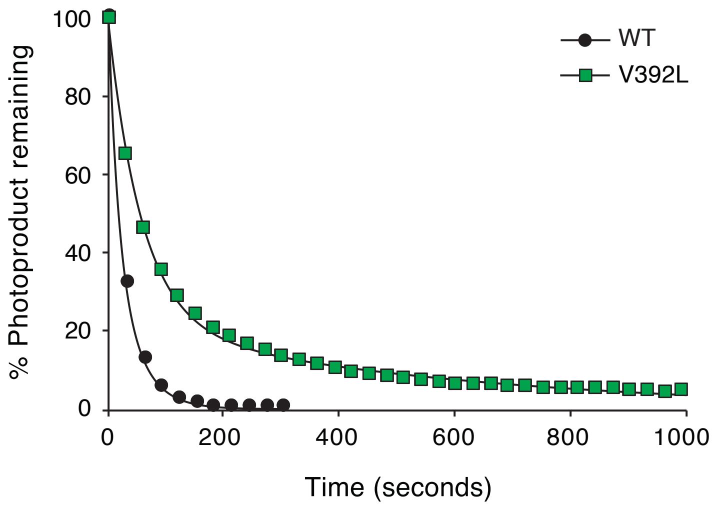

Fig. S12. Adduct decay kinetics for the slow-cycling phot2 LOV1+2 variant V392L.
Purified phot2 LOV1+2 protein was irradiated with a WL camera strobe flash and adduct decay was recorded at an absorbance of 450 nm. Decay is expressed as percentage of photoproduct remaining and was calculated from the ratio of absorbance at 450 nm obtained immediately after light exposure to that obtained for subsequent sampling in darkness. Decay was fitted to two exponentials with a half-lives of 11 and 28s for phot2 LOV1+2 (WT) and 40 and 404 s for V392L.

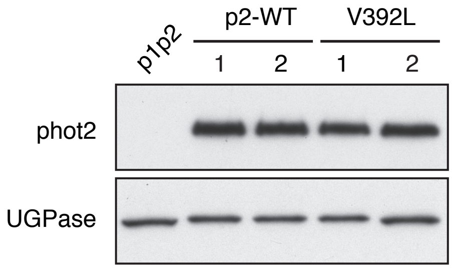

Fig. S13. Expression of phot2 and the V392L variant in the *phot1phot2* mutant of *Arabidopsis*. Immunoblot analysis of total protein extracts isolated from 3-day-old etiolated *phot1phot2* (p1p2) seedlings that express phot2-GFP (p2-WT) or the slow-cycling variant V392L. Two independent homozygous lines were examined for each genotype. Protein extracts were probed with an anti-phot2 antibody (upper panel) or an antibody raised against UDP-glucose pyrophosphorylase (UGPase) as a loading control (lower panel).

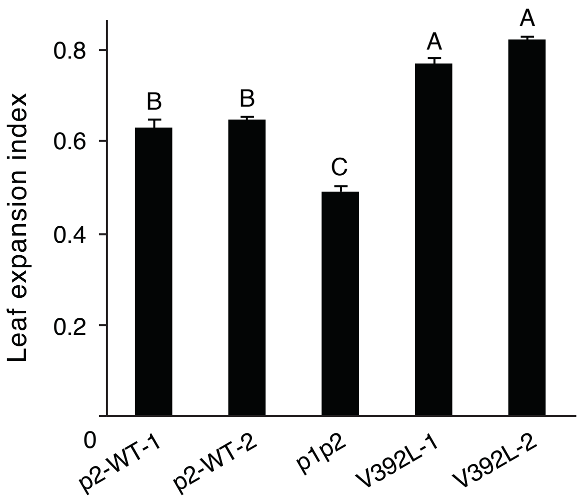

Fig. S14. Leaf expansion phenotype in *Arabidopsis* plants that express phot2 or the V392L mutant. Leaf expansion responses in *phot1phot2* (p1p2) plants that express phot2-GFP (p2-WT) or the slow photocycle mutant V392L. Two independent homozygous lines were examined for each genotype. Plants were grown for 24 days under 80 µmol m^{-2} s^{-1} white light (16/8 h L/D cycles). The leaf expansion index of the 5th rosette leaf was expressed as the ratio of the leaf area before and after artificial uncurling. Each value is the mean ± S.E. of 12 leaves. Means that do not share a letter are significantly different ($p<0.001$, one-way analysis of variance with Games-Howell post test).

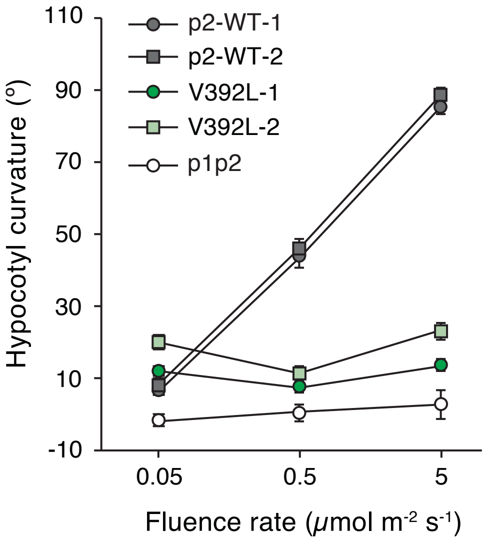

Fig. S15. Phototropic responsiveness of phot2 and the V392L mutant in etiolated seedlings. Hypocotyl phototropism in 3-day-old etiolated *phot1phot2* (p1p2) seedlings that express phot2-GFP (phot2-WT) or the slow-cycling mutant V392L. Two independent homozygous lines were examined for each genotype. Seedlings were irradiated with unidirectional BL at the fluence rates indicated for 24 h. Curvatures were calculated as the mean ± S.E. of 45-50 seedlings.

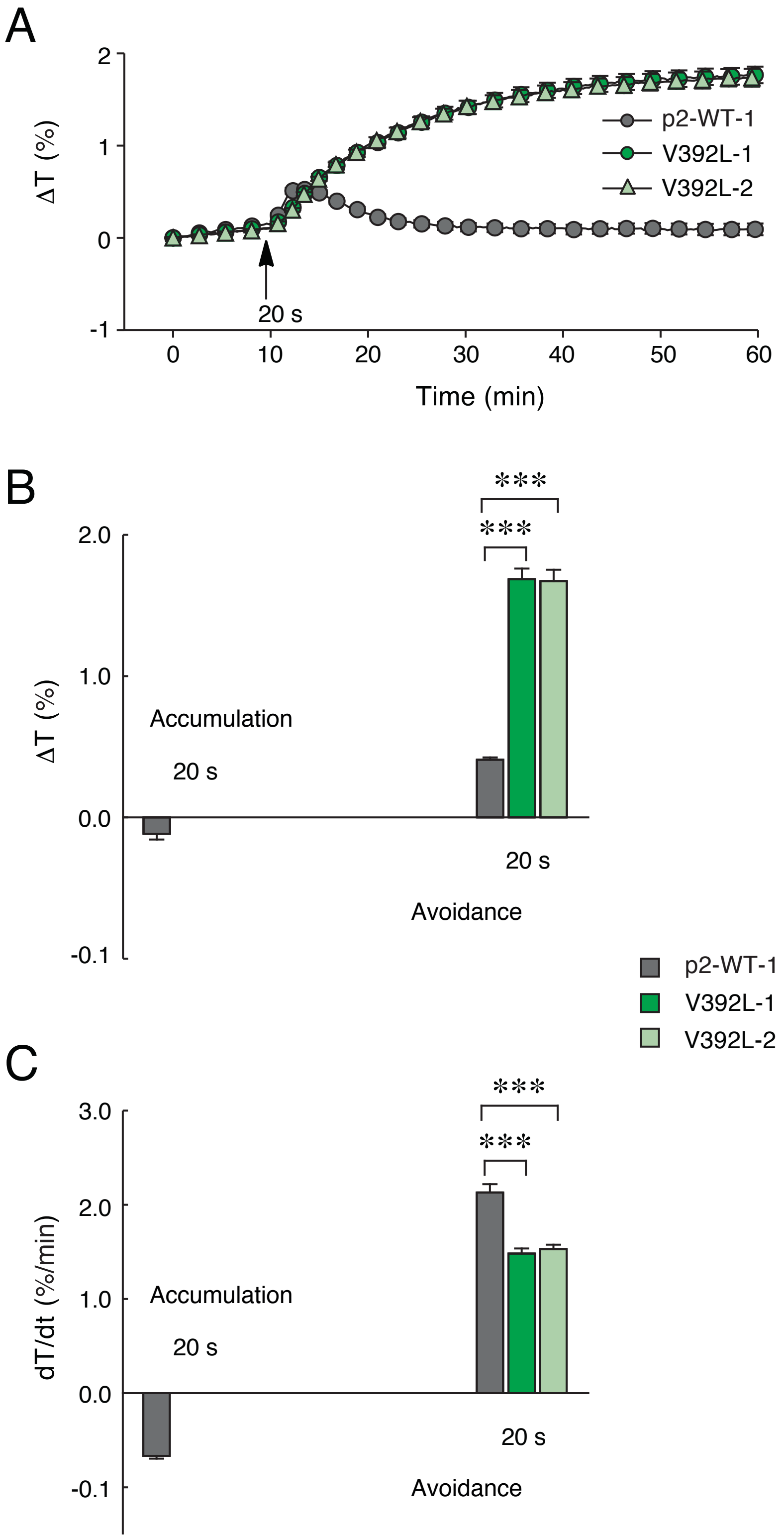

Fig. S16. Chloroplast responses in lines that express the V392L variant of phot2.
Chloroplast movement to a 20 s-long pulse of 120 µmol m^{-2} s^{-1} BL was measured in detached rosette leaves of 4-week old plants. (*A*) Time course of changes in the leaf transmittance, (*B*) amplitudes and (*C*) maximal rates of chloroplast accumulation and avoidance induced by pulses. Each value in *A*-*C* is the mean ± S.E. of 26 measurements. Asterisks indicate significant differences between photocycle mutants and the p2-WT line (***P<0.001, one-way analysis of variance with Dunnett's post test, calculated for each light dose separately).

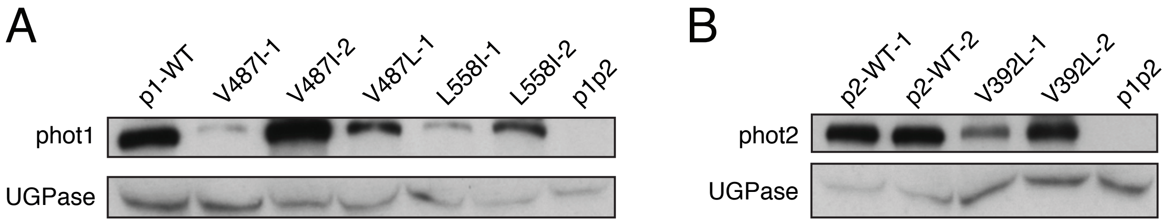

Fig. S17. Phot1 and phot2 protein abundance in light-grown seedlings.
Immunoblot analysis of total protein extracts isolated from seedlings grown under 80 µmol m^{-2} s^{-1} white light (16/8 h L/D cycles) for 9 days before being transferred to 25 µmol m^{-2} s^{-1} of RL + 0.1 µmol m^{-2} s^{-1} BL for 5 days. Protein extracts from seedlings that express (*A*) phot1-GFP (p1-WT) or the slow-cycling mutants V478I, V478L or L558I and (*B*) phot2-GFP (p2-WT) or the slow-cycling variant V392L, were probed with an anti-phot1 and anti-phot2 antibody, respectively (upper panel). An antibody raised against UDP-glucose pyrophosphorylase (UGPase) was used as a loading control (lower panel).
